# A Novel Pathogen Detection System Combining a Nucleic Acid Extraction Biochip with a Perovskite Photodetector

**DOI:** 10.3390/mi16050581

**Published:** 2025-05-15

**Authors:** Zhuo Gao, Pan Wang, Chang Chen, Jian Duan, Shilun Feng, Bo Liu

**Affiliations:** 1 School of Microelectronics, Shanghai University, Shanghai 201800, China; zhuogao@shu.edu.cn (Z.G.); chang.chen@sitri.com (C.C.); duanjian@shu.edu.cn (J.D.); 2 State Key Laboratory of Transducer Technology, Shanghai Institute of Microsystem and Information Technology, Chinese Academy of Sciences, Shanghai 200050, China; wang_pan@mail.sim.ac.cn; 3 Institute of Medical Chips, Ruijin Hospital, Shanghai Jiao Tong University School of Medicine, Shanghai 200025, China; 4 Shanghai Industrial µTechnology Research Institute, Shanghai 201800, China; 5 Jiaxing Key Laboratory of Biosemiconductors (A), Xiangfu Laboratory, Jiaxing 314102, China

**Keywords:** microfluidic chip, perovskite photodetector, fluorescence detection, nucleic acid extraction

## Abstract

The increasing spread of infectious diseases caused by pathogenic microorganisms underscores the urgent need for highly sensitive, portable, and rapid nucleic acid detection technologies to facilitate early diagnosis and effective prevention. In this study, we developed a fluorescence-based nucleic acid detection platform that integrates a microfluidic chip with an all-inorganic perovskite photodetector. The system enables integrated operation of nucleic acid extraction, purification, and amplification on a microfluidic chip, combined with real-time electrical signal readout via a CsPbBr_3_ perovskite photodetector. Experimental results indicate that the photodetector exhibits high responsivity at 530 nm, aligning well with the primary emission peak of FAM. The system demonstrates a strong linear correlation between photocurrent and FAM concentration over the range of 0.01–0.4 μM (R^2^ = 0.928), with a low detection limit of 0.01 μM and excellent reproducibility across multiple measurements. Validation using FAM standard solutions and Escherichia coli samples confirmed the system’s reliable linearity and signal stability. This platform demonstrates strong potential for rapid pathogen screening and point-of-care diagnostic applications.

## 1. Introduction

The rapid progression of globalization and urbanization has markedly increased human mobility, population density, and international connectivity, collectively contributing to the accelerated transmission of infectious diseases [1]. Under these conditions, pathogenic microorganisms—including bacteria, viruses, and other infectious agents—can spread rapidly, often demonstrating high transmissibility and acute clinical onset. These characteristics present significant challenges to global public health systems and raise the likelihood of large-scale outbreaks. In this context, the timely identification and control of pathogens are critical, underscoring the urgent need for diagnostic technologies that are not only rapid and highly sensitive but also portable and accessible in diverse settings [2].

To address these challenges, various pathogen detection strategies have been developed, with immunoassays and molecular diagnostics currently representing the most widely utilized approaches [3]. Immunoassays are commonly adopted in clinical diagnostics due to their operational simplicity, rapid results, and cost-effectiveness. However, their effectiveness in early-stage detection is often hindered by the delayed onset of the host’s immune response, particularly the time required for antibody production [4]. In contrast, molecular diagnostics—which directly target pathogen-specific nucleic acid sequences (DNA or RNA)—offer enhanced sensitivity and specificity, making them more suitable for early-stage screening and precise identification of infectious agents [5]. The continued development of nucleic acid amplification methods, such as polymerase chain reaction (PCR) and loop-mediated isothermal amplification (LAMP) [6,7], has further expanded the applicability of molecular diagnostics across multiple domains, including infectious disease surveillance, food safety monitoring, and biosafety management. To further improve the practicality and performance of molecular diagnostic platforms, microfluidic chip technology has been increasingly integrated into nucleic acid detection systems. Microfluidics offers notable advantages such as minimal sample and reagent consumption, high precision in fluid control, and strong potential for system integration, making it an ideal foundation for portable and automated diagnostic devices. Diverse detection modalities have been effectively coupled with microfluidic platforms, including electrochemical sensing [8], mechanical sensing [9], and fluorescence-based detection [10,11]. Among these, fluorescence detection stands out as the most prevalent method due to its high sensitivity, specificity, and ease of operation [12]. Conventional fluorescence detection techniques typically involve labeling amplified nucleic acids with fluorescent dyes or probes, followed by signal acquisition using fluorescence microscopy or CCD/CMOS imaging systems [13]. However, these imaging-based approaches often rely on complex image processing for quantification, which introduces subjectivity and susceptibility to human error, thereby limiting standardization and throughput.

To overcome these limitations, the direct conversion of fluorescence signals into electrical outputs has emerged as a promising strategy to enhance quantification accuracy and detection efficiency [14]. Central to this approach is the photodetector, whose performance—particularly in terms of sensitivity, response time, and spectral range—directly influences the quality of signal acquisition. Conventional photodetectors, such as photodiodes (PDs) and photomultiplier tubes (PMTs), have been extensively employed in fluorescence detection systems [15]. Despite their widespread use, PDs suffer from low sensitivity and narrow spectral bandwidth, whereas PMTs, although highly sensitive, are bulky and cost-prohibitive, making them less suitable for portable, low-cost diagnostic devices [16]. These limitations highlight the need for novel photodetection technologies optimized for point-of-care applications.

In recent years, perovskite-based sensors have attracted extensive attention from the academic community due to their outstanding optical properties, including high absorption coefficients, strong photoluminescence, excellent photoelectric conversion efficiency, and tunable bandgaps [17,18]. For example, An et al. [19] encapsulated CsPbBr_3_ perovskite nanocrystals in polystyrene/polyacrylamide microspheres to construct a urea sensor, achieving a detection limit of 1.67 μmol L^−1^ and demonstrating effective biomolecule sensing capabilities. Wang et al. [20] utilized the luminescent properties of CsPbBr_3_ in combination with the hydrophobicity of BN materials to achieve the highly sensitive detection of tetracycline, with a detection limit as low as 6.5 ng·mL^−1^. In addition, Xiang et al. [21] developed a humidity photodetector based on the water sensitivity of CsPbBr_3_ for monitoring moisture content in herbal medicines, with a minimum detection limit of 12% RH. These studies indicate that perovskite sensors not only offer high sensitivity, environmental adaptability, and stable photoelectric response, but also exhibit tunable bandgap characteristics that enable flexible spectral matching for different fluorescence signals [22]. Notably, CsPbBr_3_ shows peak responsivity around 530 nm, which closely matches the emission spectrum of the commonly used fluorophore 5(6)-Carboxyfluorescein (FAM), making it particularly suitable for direct fluorescence detection without the need for wavelength conversion or optical filtering. These advantages allow for reliable detection even in complex sample environments, highlighting the enormous potential of perovskite-based sensors in the field of fluorescence sensing [23].

This study proposes and demonstrates a nucleic acid fluorescence detection platform that integrates a microfluidic chip with an all-inorganic CsPbBr_3_ perovskite photodetector. The system incorporates nucleic acid extraction, fluorescence labeling, and signal readout into a unified workflow, enabling the efficient conversion of FAM fluorescence signals into electrical outputs and eliminating the need for traditional image-based analysis. This significantly enhances the sensitivity and portability of the detection process. The experimental results show that the photodetector exhibits excellent spectral responsivity at the main emission wavelength of FAM (520 nm), thereby improving detection specificity and photoelectric conversion efficiency. Validation using FAM standard solutions and *Escherichia coli* nucleic acid samples demonstrates the system’s favorable linearity, signal stability, and repeatability, further confirming its strong potential for rapid on-site pathogen screening.

## 2. Materials and Methods

### 2.1. Design and Construction of the Detection System

**Figure 1:**
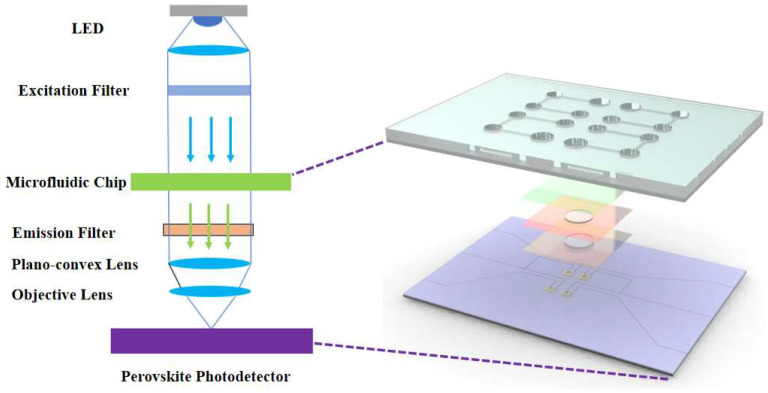
Structure of optical path in the system. The arrows in the figure indicate the direction of light propagation in the excitation and emission paths, with blue representing excitation light and green representing fluorescence signal.

To achieve highly sensitive fluorescence detection of nucleic acids within a microfluidic chip, a vertically aligned integrated optical detection system was constructed, as illustrated in Figure 1. This system employs a vertically aligned optical path comprising, from top to bottom, a fluorescence excitation module, a microfluidic chip module, a signal collection system, and a perovskite photodetector (detection module). The fluorescence excitation module consists of a 470 nm blue LED (REE, Durham, NC, USA) and an excitation filter (ET470/40x, Chroma, CA, USA), generating excitation light of a specific wavelength to stimulate fluorescent labels within the sample. The microfluidic chip module processes biological samples, including pathogen lysis, washing, and purification (see Section 2.1.2 for a detailed description of the chip structure and extraction process). The fluorescence signal collection system first employs an emission filter (ET525/50m, Chroma, CA, USA) to eliminate non-target wavelengths and stray excitation light. A plano-convex lens (f = 15 mm, JCOPTIX, Nanjing, China) collimates the divergent light into a parallel beam, which is then focused using a microscope objective lens (0.45 NA, 20×, 26.5 mm, Olympus, Tokyo, Japan) onto the photosensitive area of the perovskite photodetector (Figure 1). Finally, the perovskite photodetector converts the fluorescence signal into an electrical signal for digital output.

#### 2.1.1. Structure and Working Principle of the Perovskite Photodetector

**Figure 2:**
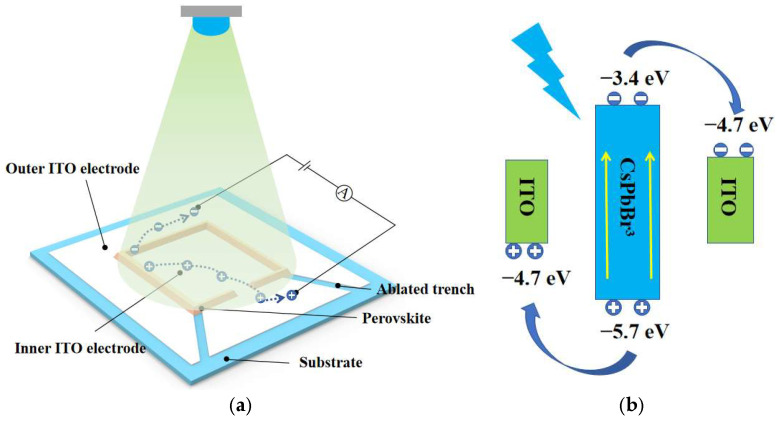
(**a**) Schematic diagram of the working principle of the perovskite photodetector; (**b**) schematic energy band diagram of the CsPbBr_3_ layer and ITO electrodes.

The structure of the perovskite photodetector developed in this study is shown in Figure 2a. Nanosecond pulsed laser ablation was employed to precisely define the conductive pathways of the ITO layer. As illustrated in Figure 1, under focused laser irradiation, the surface of the ITO was gradually ablated, and the irradiated regions of the ITO film were completely removed, thereby exposing the underlying glass substrate. Owing to the excellent conductivity of the ITO layer, this laser ablation process created electrical isolation in the trench regions, effectively dividing the ITO into two separate regions: an inner electrode located within the perovskite-patterned area and an outer electrode surrounding it. During the ablation process, the electrode layout was carefully optimized to maximize the contact area between the perovskite active region and the outer electrode, thereby enhancing charge transport efficiency and improving the device’s photoelectric conversion performance. A CsPbBr_3_ perovskite thin film was subsequently deposited in the patterned region, forming a bridging structure between the inner and outer electrodes. Upon illumination, the perovskite layer absorbs incident photons and generates electron–hole pairs. Under the applied external bias, the photogenerated electrons and holes are driven toward their respective electrodes, resulting in a measurable photocurrent in the external circuit. Figure 2b further explains the energy band structure and carrier transport pathways under illumination. When the energy of the incident photons exceeds the bandgap of CsPbBr_3_, electrons in the valence band are excited to the conduction band, forming electron–hole pairs. The excited electrons occupy conduction band energy levels near −3.4 eV and tend to migrate toward the right ITO electrode, which provides favorable energy alignment for efficient electron injection and collection. Simultaneously, the holes stay in the valence band (approximately −5.7 eV) and migrate toward the left ITO electrode, whose work function (approximately −4.7 eV) is energetically favorable for hole collection. When the external circuit is closed, electrons flow from the right ITO electrode to the left through the external circuit and recombine with holes at the left ITO electrode, thereby generating a stable photocurrent.

#### 2.1.2. Design of the Microfluidic Chip and Process of Nucleic Acid Lysis and Purification

**Figure 3:**
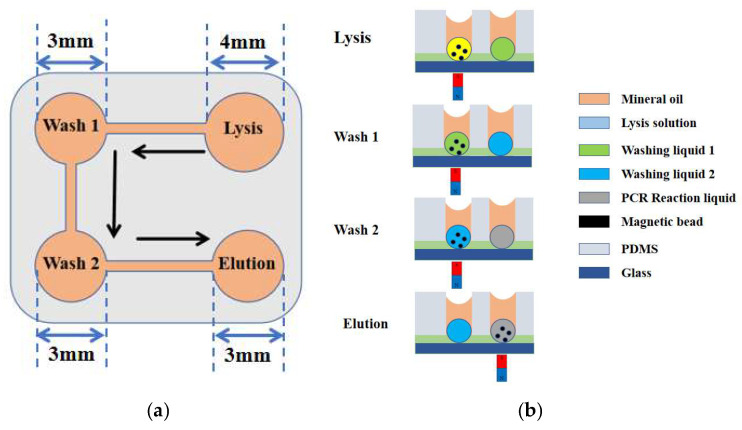
(**a**) Schematic layout of the microfluidic chip designed for nucleic acid extraction and purification, the arrows indicate the directional path of magnetic bead movement; (**b**) the detection process, from sample loading to elution, is shown as a side view in the sequence of lysis, wash 1, wash 2, elution, and amplification.

As illustrated in Figure 3a, the microfluidic chip for nucleic acid extraction based on magnetic bead-based methodology [24] consists of four functional chambers: the lysis chamber, the first washing chamber, the second washing chamber, and the elution chamber. These chambers are sequentially used for pathogen lysis, primary washing, secondary washing, and nucleic acid elution using magnetic beads, respectively. The detailed extraction and purification workflow is shown in Figure 3b. Initially, pathogens were lysed in the lysis chamber by the action of a lysis buffer, resulting in the release of nucleic acids, which were subsequently adsorbed onto the surface of magnetic beads via electrostatic interactions. Then, the magnetic beads carrying the nucleic acids were transported by an external magnetic field through the first and second washing chambers to remove impurities generated during the lysis process. Finally, nucleic acids were eluted from the magnetic beads using an elution buffer in the elution chamber.

The height of each chamber was set to 4 mm. The diameters of the lysis, first washing, second washing, and elution chambers were 4 mm, 3 mm, 3 mm, and 3 mm, respectively. The microchannels connecting the chambers had a width of 0. 8 mm and a height of 100 μm.

### 2.2. Experimental Section

#### 2.2.1. Reagents and Materials

This study involves the fabrication of perovskite photodetectors, microfluidic chips, and their application in biological detection. The main reagents and materials used are as follows: for the fabrication of the perovskite photodetector, cesium bromide (CsBr) and lead (II) bromide (PbBr_2_) (both purchased from Aladdin Reagent Co., Ltd., Shanghai, China) were used as precursor materials. Indium tin oxide-coated conductive glass (ITO-glass, Shangyang Solar Technology, Suzhou, China) served as the substrate, and the surface was modified using 1H, 1H, 2H, 2H-perfluorooctyltriethoxysilane (POTS, Sigma-Aldrich, St. Louis, MO, USA). For the microfluidic chip fabrication, SU-3050 photoresist (Sigma-Aldrich, St. Louis, MO, USA) and 4-inch single-crystal silicon wafers (Shanghai Institute of Microsystem and Information Technology, Shanghai, China) were used. Polydimethylsiloxane (PDMS) and its corresponding curing agent (Momentive Performance Materials, Wilton, CT, USA), along with standard glass slides (Thermo Fisher Scientific, Waltham, MA, USA), were employed for replica molding to form the microfluidic channels. During biological detection, 5(6)-carboxyfluorescein (FAM, Titan Scientific, Shanghai, China) and dimethyl sulfoxide (DMSO, Aladdin Reagent, Shanghai, China) were used to prepare the fluorescent solutions. Silanol-coated magnetic beads for nucleic acid extraction (average particle size of 500 nm, Sangon Biotech, Shanghai, China), lysis buffer (Sansure Biotech, Changsha, China), absolute ethanol (Shanghai Lingfeng Chemical Reagent, Shanghai, China), and polyethylene glycol-8000 (PEG-8000, Beijing Dingguo Biotechnology Development Center, Beijing, China) were used for nucleic acid lysis and purification. In addition, high-purity mineral oil (Sigma-Aldrich, St. Louis, MO, USA) was used as an auxiliary reagent. Double-distilled water (ddH_2_O) was employed as the primary solvent throughout the experimental procedures.

#### 2.2.2. Fabrication of Perovskite Photodetector Based on CsPbBr_3_

To precisely control the wettability contrast between hydrophilic and hydrophobic regions, plasma treatment and POTS modification were applied to selectively modify the ITO surface. This approach significantly enhanced the wettability contrast between regions, enabling the CsPbBr_3_ precursor solution to selectively localize within the hydrophilic patterned areas, thereby promoting ordered crystallization and the formation of high-quality perovskite thin films.

During the fabrication of the perovskite photodetector, ITO-glass was used as the substrate. The substrate was sequentially cleaned by ultrasonic treatment in ethanol and deionized water (5 min each), followed by nitrogen drying to remove surface impurities. Oxygen plasma treatment (2 min) was then applied to enhance surface activity and facilitate functionalization. The treated ITO substrate was placed in a sealed container with 2 μL of POTS and heated at 120 °C for 2.5 h to form a hydrophobic modification layer. Selective ablation was performed on the functionalized substrate using a nanosecond pulsed laser (λ = 355 nm, 50 kHz, 1.045 W) to generate predefined regions for selective deposition of the perovskite precursor. Lead bromide (PbBr_2_) and cesium bromide (CsBr) were dissolved in DMSO at a molar ratio of 1:1 and ultrasonicated for 4 h to achieve complete dispersion, forming a 0.4 mol·L^−1^ precursor solution. The precursor was uniformly spin-coated onto the hydrophilic regions, followed by pre-crystallization on a hot plate at 60 °C and subsequent annealing in a vacuum oven at 80 °C for 8 h to yield highly crystalline CsPbBr_3_ films. Finally, laser ablation was employed to structure the ITO electrodes for functional separation.

#### 2.2.3. Fabrication of the Microfluidic Chip

**Figure 4:**
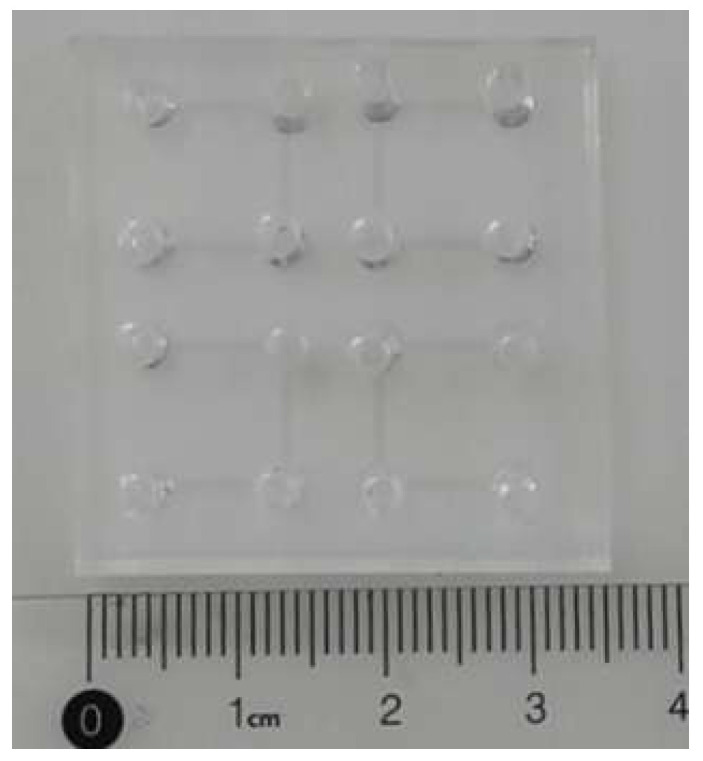
Image of the fabricated microfluidic chip.

The microfluidic chip used in this study was fabricated using a maskless photolithography technique. First, SU-8 3050 negative photoresist was spin-coated onto a silicon wafer, followed by patterning exposure using a UV lithography system (MicroWriter, Durham Magneto Optics, Cambridge, UK). After development, a silicon-based mold with the desired microstructures was obtained. Subsequently, polydimethylsiloxane (PDMS) prepolymer and curing agent were mixed in a 10:1 weight ratio and degassed under vacuum to remove air bubbles. The degassed mixture was then poured onto the silicon mold and cured at 65 °C for 4 h. After curing, the PDMS layer was peeled off, and through-holes were created at designated positions using a biopsy punch. Finally, the PDMS layer was plasma-bonded to a glass substrate via plasma treatment to form the nucleic acid extraction microfluidic chip. The assembled chip was then placed in an oven at 65 °C for 12 h to further enhance the bonding strength at the PDMS–glass interface. A photographic image of the fabricated microfluidic chip is shown in Figure 4.

#### 2.2.4. On-Chip Nucleic Acid Detection Process

To initiate the nucleic acid detection process, 200 μL of mineral oil was injected into the microfluidic chip to ensure complete filling of all functional chambers. Subsequently, the following reagents were introduced into their corresponding chambers: 8 μL of lysis buffer (containing 7.5 μL extraction reagent, 0.25 μL proteinase K, and 0.5 μL silica-coated magnetic beads); 4 μL of first washing buffer (70% ethanol); 4 μL of second washing buffer (13% PEG-8000); and 4 μL of PCR amplification reagent. This configuration generated a reaction droplet, two washing droplets, and a detection droplet within the chip. Then, 5 μL of the biological sample was added to the lysis chamber. A magnet was employed to facilitate the mixing of reagents and the collection of magnetic beads. The mixture was incubated in the lysis chamber for 2 min with active mixing, followed by 10 min of static incubation. The magnetic beads, now bound to nucleic acids, were transferred through the first and second washing chambers, where they were mixed for 1 min in each chamber to enhance purification. Finally, the beads were moved into the amplification chamber, mixed for 1 min, and incubated for 5 min before being magnetically returned to the previous chamber for further processing. After sample processing, the microfluidic chip was sealed with PCR sealing film and placed in an on-chip thermal cycler for nucleic acid amplification.

## 3. Results and Discussion

### 3.1. Photoelectric Properties of the CsPbBr_3_ Photodetector

The performance of the photodetector was evaluated in a controlled dark environment. A signal generator (AFG3251C, Tektronix, Beaverton, OR, USA) was used to power a blue LED, which served as the excitation light source, while a semiconductor parameter analyzer (4200A−SCS, Keithley, Cleveland, OH, USA) was employed to measure the photoelectric response of the device. A bias voltage of 5 V was applied to the perovskite photodetector during signal acquisition process.

**Figure 5:**
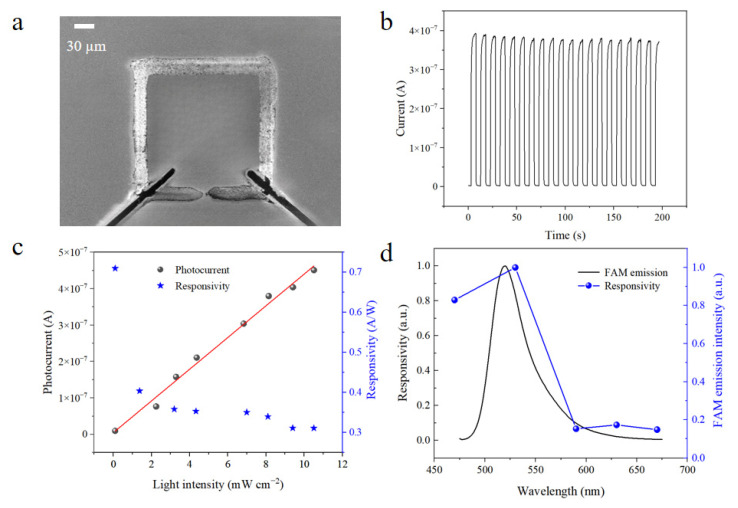
Performance characterization of a CsPbBr_3_ perovskite photodetector for FAM fluorescence sensing. (**a**) SEM image of the CsPbBr_3_ perovskite photodetector; (**b**) on/off switching stability test of the device under a 530 nm pulsed light source at a frequency of 0.01 Hz; (**c**) photocurrent and responsivity of the photodetector under different light intensities at 530 nm; (**d**) relative photoresponse of the perovskite photodetector across different wavelengths, along with the emission spectrum of FAM.

Figure 5a presents the scanning electron microscopy (SEM) image of the perovskite photodetector fabricated using CsPbBr_3_ material. The device features an insulating trench formed by laser ablation, which effectively separates the ITO electrode regions and enables lateral current flow between the internal and external regions. This structural design significantly shortens the carrier transport path and enhances the utilization efficiency of excitation light within the photoactive perovskite layer. Figure 5b displays the time-resolved photoresponse curve of the device under periodic illumination at 530 nm. Stable and highly repeatable photocurrent pulse responses were clearly observed, with consistent peak and baseline levels maintained over multiple excitation cycles. These results indicate excellent device stability and durability under continuous illumination, which is essential for real-time monitoring and practical applications. This further validates the reliable performance of the CsPbBr_3_-based photodetector.

Figure 5c shows the photocurrent response of the device under 530 nm illumination at varying light intensities of illumination. As the incident power density increased from 0.01 mW cm^−2^ to 10.24 mW cm^−2^, the photocurrent exhibited linear growth, indicating good linearity over a broad range of illumination intensities. This behavior can be explained by the photogeneration of electron–hole pairs [25]: as light intensity increases, more photons are absorbed by the perovskite film, leading to the generation of more photocarriers and, consequently, enhanced photocurrent output. The figure also shows the device responsivity (defined as the ratio of photocurrent to incident optical power). A decreasing trend in responsivity is observed with increasing light intensity, which can be attributed to enhanced carrier recombination rates [26] that limit further photocurrent increase, resulting in a sublinear responsivity behavior. Figure 5d illustrates the wavelength-dependent responsivity of the device along with the emission spectrum of FAM fluorescent dye (See Supplementary Materials for details). The CsPbBr_3_ photodetector exhibits a peak responsivity at 530 nm, which closely matches the primary emission wavelength of FAM. This excellent spectral alignment confirms the device’s capability for efficient FAM fluorescence detection, providing a solid foundation for high-sensitivity signal acquisition in nucleic acid detection systems.

### 3.2. Linearity Test of FAM Fluorophore Detection

To evaluate the performance of the perovskite-based fluorescence detection system, FAM fluorescent solutions were prepared by first dissolving 7.52 mg of 5(6)-carboxyfluorescein in 1 mL of DMSO to obtain a 0.1 mol L^−1^ stock solution. This stock solution was subsequently diluted with ddH_2_O to obtain working solutions with concentrations of 0.01 μM, 0.05 μM, 0.1 μM, 0.2 μM, 0.3 μM, and 0.4 μM. During fluorescence detection, 10 μL of the prepared FAM solution was injected into the microfluidic chip, and a pulsed light source with a cycle period of 10 s and a duty cycle of 50% was employed to excite the fluorescent molecules. The photocurrent response generated by the perovskite photodetector was then recorded to assess the system’s sensitivity and signal stability under varying FAM concentrations. All experiments were conducted at room temperature (approximately 25 °C) to ensure thermal consistency and eliminate temperature-related signal variations.

**Figure 6:**
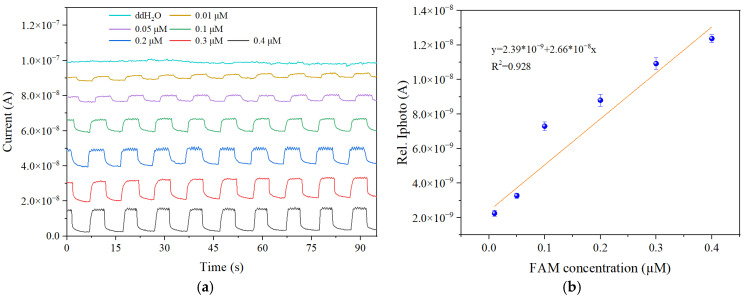
(**a**) Photocurrent responses of the perovskite photodetector to FAM fluorophore solutions at varying concentrations; (**b**) linear fitting curve of the relative photocurrent values as a function of FAM concentration.

Figure 6a presents the current response curves corresponding to different concentrations of FAM solutions (0 μM, 0.01 μM, 0.05 μM, 0.1 μM, 0.2 μM, 0.3 μM, and 0.4 μM). Initially, ddH_2_O was used as a control to establish the baseline. The results indicated that the photodetector exhibited negligible current fluctuations in the absence of fluorescein, suggesting a near-zero baseline response and excellent background suppression. Subsequently, the current response was measured with increasing FAM concentrations, starting at 0.01 μM. The experimental results demonstrated that the current signal was noticeably enhanced at 0.01 μM, significantly exceeding the baseline noise level. This indicates that the photodetector was able to generate a detectable photoresponse at this low concentration. It is worth emphasizing that all fluorescence detection experiments across the full concentration range shown in Figure 6a were conducted using the same CsPbBr_3_ perovskite photodetector, without any observable performance degradation over repeated excitation cycles and varying FAM concentrations. This result clearly demonstrates the excellent reusability of the perovskite photodetector, indicating its stability and practical feasibility for repeated or continuous fluorescence detection tasks in real-world applications. A higher sensitivity implies a greater ability to detect subtle changes in biomolecular concentration [27,28]. When the FAM concentration increased from 0.01 μM to 0.4 μM, the photocurrent exhibited a change of more than 10 nA, corresponding to a sensitivity exceeding 25.6 nA μM^−1^. This indicates that the sensor can generate a distinct and measurable signal amplitude response to changes in analyte concentration, demonstrating excellent responsiveness to weak fluorescence signals and fulfilling the requirements for detecting low concentrations of nucleic acids or biomarkers.

To obtain accurate response values, the current data during each excitation phase and dark stabilization phase were averaged, and the difference between the two was used to extract the effective photoresponse signal (as shown in Figure 6b). Within the FAM concentration range of 0.01 μM to 0.4 μM, the system exhibited a strong linear relationship between photocurrent and concentration. The linear fitting equation was y = 2.39 × 10^−9^ + 2.66 × 10^−8^ *x*, with a correlation coefficient of R^2^ = 0.928, indicating strong linearity.

For each concentration tested in the experiment, the signal-to-noise ratio (SNR) was calculated using Equation (1).

(1) $$\mathrm{SNR}=20\;{log}_{10}\left(\frac{I_{light}-I_{dark}\;}{2\sigma_{light}}\right)$$

where *I*_light_ and *I*_dark_ represent the average photocurrents under illumination and in darkness, respectively, and $\sigma_{light}$ denotes the standard deviation of the photocurrent under illumination. Based on the experimental data, the system achieved SNR values exceeding 20 dB starting from a concentration of 0.05 μM. Even at the lowest tested concentration of 0.01 μM, the system maintained a detectable signal with an SNR of 16.44 dB, which is above the standard threshold of 10 dB required for reliable quantitative detection. This confirms that the signal is distinguishable from background noise and can be used for low-concentration fluorescence signal identification and preliminary quantification.

Based on the criterion that the photocurrent signal must be significantly higher than the background noise, the experimental limit of detection (LoD) for the system was determined to be 0.01 μM. Within the concentration range of 0.01–0.4 μM, the system exhibited a strong linear correlation between photocurrent and FAM concentration, confirming the effective detection capability, high sensitivity, and good quantification performance of the developed perovskite-based detection platform. In future work, further improvements in detection precision may be achieved by optimizing the optical path, minimizing ambient light interference, and precisely adjusting lens focal distances.

### 3.3. Results and Analysis of Pathogen Detection

Biological application experiments are the most reliable and crucial means of evaluating the performance of a detection system. Based on the previously described PCR protocol, nucleic acid amplification was carried out using *Escherichia coli* nucleic acid at a concentration of 100 copies·μL^−1^ as the positive sample. Unamplified samples, negative samples, and ddH_2_O were used as control groups. After amplification, all samples were tested using both a commercial fluorescence microscope and the fluorescence detection system developed in this study. The detection procedure followed the same protocol as that employed in the FAM solution experiments.

**Figure 7:**
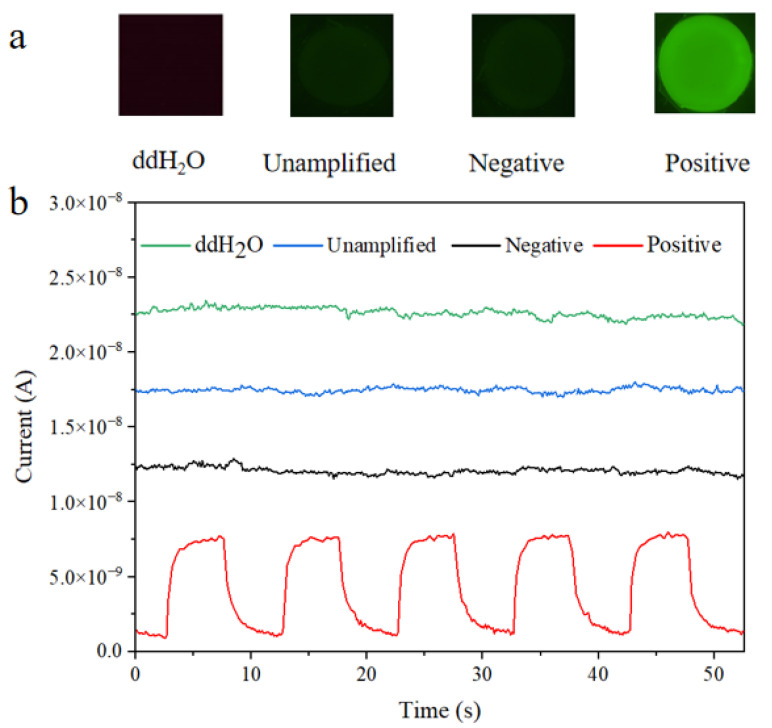
(**a**) Fluorescence imaging of the tested samples: ddH_2_O, unamplified sample, negative sample, and positive sample (from left to right); (**b**) time-resolved output current curves measured by the fluorescence detection system.

Figure 7a shows the fluorescence microscopy images of the tested samples, arranged from left to right: ddH_2_O, unamplified sample, negative sample, and positive sample. A distinct green fluorescence signal was observed in the positive sample, whereas the negative, unamplified, and ddH_2_O control samples exhibited only weak background fluorescence. This residual signal is attributed to nonspecific fluorescence from primers and probes. Figure 7b presents the time-resolved output current curves obtained from the perovskite-based fluorescence detection system. From bottom to top, the curves correspond to the positive, negative, unamplified, and ddH_2_O control samples, respectively. Under blue light excitation, the positive sample produced a pronounced pulsed photocurrent response, whereas the other three control samples showed minimal current variation and no significant photoresponse. The trend in photocurrent output closely mirrored the fluorescence intensity observed in the microscope images, further confirming the system’s sensitivity and reliability in detecting fluorescence signals from PCR-amplified nucleic acids.

## 4. Conclusions

This study proposes and validates a fluorescence-based nucleic acid detection platform that integrates a microfluidic chip with an all-inorganic perovskite photodetector, providing a novel and practical solution for rapid pathogen detection. The platform enables automated microscale nucleic acid extraction and amplification, followed by efficient fluorescence signal acquisition and quantification through a CsPbBr_3_ perovskite photodetector. This study proposes and validates a fluorescence-based nucleic acid detection platform that integrates a microfluidic chip with an all-inorganic perovskite photodetector, providing a novel and practical solution for rapid pathogen detection. The platform enables automated microscale nucleic acid extraction and amplification, followed by efficient fluorescence signal acquisition and quantification through a CsPbBr_3_ perovskite photodetector. The experimental results show that the CsPbBr_3_ detector exhibits peak responsivity at 520 nm, corresponding to the main emission wavelength of FAM. The system demonstrates a strong linear correlation between photocurrent and FAM concentration over the range of 0.01–0.4 μM (R^2^ = 0.928), with a low detection limit of 0.01 μM and excellent reproducibility across multiple measurements. Further validation using Escherichia coli nucleic acid samples confirms that the electrical signal output can accurately distinguish between positive and negative specimens, with the results highly consistent with fluorescence microscopy. A key innovation of this work lies in the integration of perovskite materials into a fluorescence detection system. The approach supports low-cost fabrication and leverages the tunable bandgap properties of perovskites to achieve spectral alignment with various fluorophores such as FAM and 6-Carboxy-X-Rhodamine (ROX), laying the foundation for future multiplexed detection. In addition, the micro/nanofabrication process of the perovskite photodetector enables microscale array layouts, offering a promising pathway toward high-throughput, multi-target, and miniaturized diagnostic devices. In summary, the integrated microfluidic–perovskite detection platform features high sensitivity, digital signal output, low cost, and strong scalability, making it particularly well-suited for rapid pathogen screening and point-of-care diagnostics in resource-limited settings.

## Data Availability

The data presented in this study are available from the corresponding authors upon reasonable request.

## Supplementary Materials

The following supporting information can be downloaded at: https://www.chroma.com, Figure 5d.
